# Production and Anti-Melanoma Activity of Methoxyisoflavones from the Biotransformation of Genistein by Two Recombinant *Escherichia coli* Strains

**DOI:** 10.3390/molecules22010087

**Published:** 2017-01-04

**Authors:** Chien-Min Chiang, Yu-Jhe Chang, Jiumn-Yih Wu, Te-Sheng Chang

**Affiliations:** 1 Department of Biotechnology, Chia Nan University of Pharmacy and Science, No. 60, Sec. 1, Erh-Jen Rd., Jen-Te District, Tainan 71710, Taiwan; cmchiang@mail.cnu.edu.tw; 2 Department of Biological Sciences and Technology, National University of Tainan, No. 33, Sec. 2, Shu-Lin St., Tainan 70005, Taiwan; roger199382@yahoo.com.tw; 3 Institute of Biotechnology and Chemical Engineering, I-Shou University, No. 1, Sec. 1, Syuecheng Rd., Dashu District, Kaohsiung 84001, Taiwan; 4 Center for General Education, National Quemoy University, No. 1, University Road., Jin-Ning Township, Kinmen County 892, Taiwan

**Keywords:** genistein, melanoma, methoxyisoflavone, methyltransferase, tyrosinase

## Abstract

Biotransformation of the soy isoflavone genistein by sequential 3′-hydroxylation using recombinant *Escherichia coli* expressing tyrosinase from *Bacillus megaterium* and then methylation using another recombinant *E. coli* expressing *O*-methyltransferase from *Streptomyces peucetius* was conducted. The results showed that two metabolites were produced from the biotransformation, identified as 5,7,4′-trihydroxy-3′-methoxyisoflavone and 5,7,3′-trihydroxy-4′-methoxyisoflavone, respectively, based on their mass and nuclear magnetic resonance spectral data. 5,7,4′-Trihydroxy-3′-methoxyisoflavone showed potent antiproliferative activity toward mouse B16 melanoma cells with an IC_50_ value of 68.8 μM. In contrast, the compound did not show any cytotoxicity toward mouse normal fibroblast cells, even at 350 μM concentration. The results of the present study offer insight on the production of both 5,7,4′-trihydroxy-3′-methoxyisoflavone and 5,7,3′-trihydroxy-4′-methoxyisoflavone by two recombinant *E. coli* strains and the potential anti-melanoma applications of 5,7,4′-trihydroxy-3′-methoxyisoflavone.

## 1. Introduction

Isoflavones are a kind of flavonoid widely distributed in several plants, such as red clover and soybeans [1,2]. Genistein and daidzein are the two major isoflavone aglycones found in soybeans. These two isoflavones have received much attention over the past few decades due to their possible roles in preventing certain hormone-dependent diseases [3]. In recent years, modification of soy isoflavones using gene-engineered microorganisms has also been of interest, as the bioactivity of compounds with different structures is dramatically altered [4].

Hydroxylation and methylation are two common modifications of flavonoids occurring in Nature. Both modifications produce more complex flavonoids, which sometimes possess higher bioactivity than their precursors. For conducting such modifications in laboratories, researchers have constructed several gene-engineered microorganisms. Using genetically-modified microorganisms to perform biotransformations has the advantage of allowing the rational design of desirable biotransformation products. Moreover, some microorganisms, such as the bacterium *E. coli* or the yeast *Pichia pastoris*, are easily handled in both biotransformation and scale-up processes [5]. Therefore, using genetically modified microorganisms to carry out biotransformations of isoflavones is an interesting issue. 

Recently, Lee et al. used a recombinant *E. coli* expressing tyrosinase from *Bacillus megaterium* to catalyze the *ortho*-hydroxylation of daidzein and genistein in the presence of borate and ascorbic acid [6]. The biotransformation system was also successfully applied to catalyze the *ortho*-hydroxylation of the soy isoflavone glycosides daidzin and genistin [7]. On the other hand, a recombinant *E. coli* expressing *O*-methyltransferase from *Streptomyces peucetius* was demonstrated to catalyze the *O*-methylation of *ortho*-dihydroxyisoflavone [8]. Based on the results of these previous studies, it is possible to use the two recombinant *E. coli* strains to conduct dual modifications, including hydroxylation and *O*-methylation, on soy isoflavones. In the present study, biotransformation of the soy isoflavone genistein, first by 3′-hydroxylation using the recombinant *E. coli* expressing *B. megaterium* tyrosinase and second by *O*-methylation using the recombinant *E. coli* expressing *S. peucetius O*-methyltransferase, was performed, and the anti-proliferative activity of the resulting biotransformation metabolites on both mouse melanoma and normal fibroblast cells was also determined.

## 2. Results

### 2.1. Biotransformation of Genistein by the Two Recombinant E. coli Strains and Purification and Identification of the Biotransformation Products

**Figure 1:**
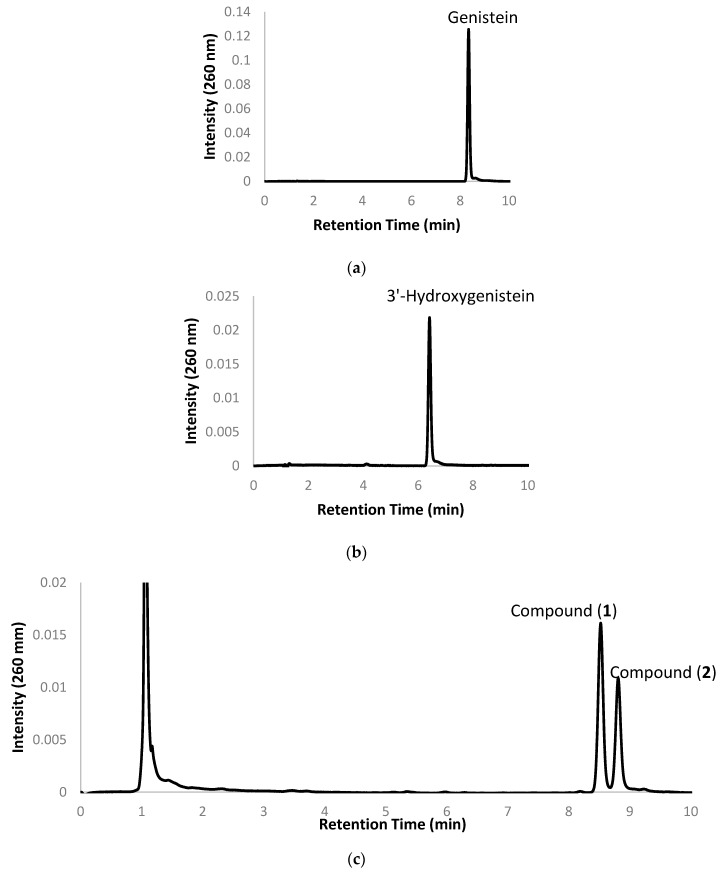
UPLC profiles of the biotransformation precursor genistein (**a**); the products of the first biotransformation reaction using the recombinant *E. coli* expressing tyrosinase (**b**); the products of the second biotransformation reaction using the recombinant *E. coli* expressing *O*-methyltransferase (**c**). The detailed protocols for biotransformation and UPLC are described in Materials and Methods.

In the present study, commercial genistein soy isoflavone was used as a biotransformation precursor, which appeared at retention time of 8.34 min in the ultra-performance liquid chromatography (UPLC) analysis (Figure 1a). Figure 1b shows the UPLC profile of the biotransformation product of genistein using recombinant *E. coli* expressing *B. megaterium* tyrosinase in the presence of 500 mM of borate (pH 9.0) and 10 mM of ascorbate. As shown in the figure, the genistein peak almost completely disappeared and a new peak appeared at a retention time of 6.42 min. According to the results by Lee et al. [6] and our previous study [7], the new peak at 6.42 min should be 3′-hydroxygenistein. In the biotransformation, genistein was almost completed converted. The result is consistent with that of Lee et al., who showed that a 93% conversion yield was achieved using the recombinant *E. coli* for 3′-hydroxylation of genistein [6].

In a second biotransformation, the produced 3′-hydroxygenistein from the first biotransformation reaction was incubated with the recombinant *E. coli* expressing *S. peucetius O*-methyltransferase in the presence of 1 mM of *S*-adenosylmethionine (SAM) for 24 h. Figure 1c shows the UPLC profile of the second biotransformation reaction product. In the figure, two new peaks appear—compounds (**1**) and (**2**), with retention times of 8.52 and 8.81 min, respectively. The results revealed that the 3′-hydroxygenistein produced by the first biotransformation reaction was further biotransformed in the second biotransformation reaction.

The metabolites of the second reaction were isolated using a preparative high-performance liquid chromatography (HPLC) method and were identified using spectrophotometric methods. Compound **1** showed an [M + H]^+^ ion peak at *m*/*z*: 301 in the electrospray ionization mass (ESI-MS) spectrum, corresponding to the molecular formula C_16_H_12_O_6_. Then both ^1^H and ^13^C nuclear magnetic resonance (NMR) experiments including heteronuclear multiple quantum coherence (HMQC), heteronuclear multiple bond connectivity (HMBC), nuclear Overhauser effect spectroscopy (NOESY), and correlation spectroscopy (COSY) spectra were performed and the ^1^H- and ^13^C-NMR signal assignments were conducted accordingly. The HMBC spectrum revealed a methoxyl proton signal at δ 3.79(s) correlated to carbon resonance at δ 147.3 (C-3′), and the NOESY spectrum revealed the distance proximity between protons at δ 3.79(s) and protons at δ 7.13 (d, H-2′). Based on these spectral data and comparison with the ^1^H-NMR and ^13^C-NMR data in the literature [9], compound **1** was characterized as 5,7,4′-trihydroxy-3′-methoxyisoflavone.

**Scheme 1:**
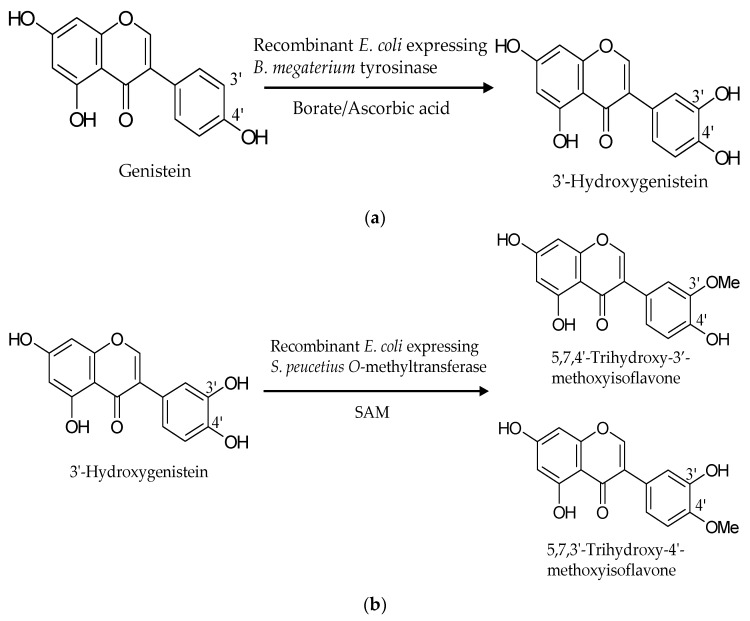
Diagram of the biotransformation of genistein by the two recombinant *E. coli*. (**a**) Genistein was firstly biotransformed to 3′-hydroxygenistein by recombinant *E. coli* that expressed *B. megaterium* tyrosinase; (**b**) The produced 3′-hydroxygenistein was then biotransformed to 5,7,4′-trihydroxy-3′-methoxyisoflavone and 5,7,3′-trihydroxy-4′-methoxyisoflavone by recombinant *E. coli* that expressed *S. peucetius O*-methyltransferase.

Compound **2** showed an [M + H]^+^ ion peak at *m*/*z*: 301. The HMBC spectrum revealed a methoxyl proton signal at δ 3.79(s) correlated to the carbon resonance at δ 157.4 (C-4′), and the NOESY spectrum revealed the distance proximity between protons at δ 3.79(s) and at δ 7.02 (d, H-5′). Based on these spectral data and a comparison of the ^1^H-NMR and ^13^C-NMR data with literature values [10], compound **2** was characterized as 5,7,3′-trihydroxy-4′-methoxyisoflavone. These results—together with our previous study [7,8] and the study performed by Lee et al. [6]—reveal that genistein was first converted into 3′-hydroxygenistein by the recombinant *E. coli* expressing *B. megaterium* tyrosinase, and then the resulting 3′-hydroxygenistein was further transformed by recombinant *E. coli* expressing *S. peucetius O*-methyltransferase into 5,7,4′-trihydroxy-3′-methoxyisoflavone or 5,7,3′-trihydroxy-4′-methoxyisoflavone. Scheme 1 shows a diagram of the biotransformation of genistein by the two recombinant *E. coli* strains.

### 2.2. Anti-Melanoma Activity of the Biotransformation Products

The search for new melanogenesis inhibitors for use in skin-whitening cosmetics is an interesting topic [8,11,12]. In the assay for evaluating melanogenesis inhibitory activity, mouse B16 melanoma cells are used [12,13]. After producing the two methoxyisoflavones in the present study, the melanogenesis inhibitory activity of the two methoxyisoflavones were determined by culturing B16 melanoma cells with them. To our surprise, the two methoxyisoflavones were found to be highly toxic to the melanoma cells. Table 1 shows the cytotoxicity experiment results. Among the tested compounds, 5,7,4′-trihydroxy-3′-methoxyisoflavone exhibited the most potent cytotoxicity toward the B16 melanoma cells, with an IC_50_ value of 68.1 μM. To evaluate the selectivity of the anti-melanoma activity, the cytotoxicity experiments were performed again using normal mouse fibroblast cells. The results showed that 5,7,4′-trihydroxy-3′-methoxyisoflavone exhibited no significant cytotoxicity toward mouse normal fibroblast cells, even at 350 μM concentration (Table 1). In addition, the biotransformation precursor genistein also showed potent cytotoxicity toward B16 melanoma cells, with an IC_50_ value of 70.3 μM, but little cytotoxicity toward mouse normal fibroblast cells (Table 1).

## 3. Discussion

The present study successfully developed a two-step biotransformation process for the production of 5,7,4′-trihydroxy-3′-methoxyisoflavone (**1**) and 5,7,3′-trihydroxy-4′-methoxyisoflavone (**2**) first using recombinant *E. coli* expressing *B. megaterium* tyrosinase and then using recombinant *E. coli* expressing *S. peucetius O*-methyltransferase. To our knowledge, this is the first report of the bio-production of the two methoxyisoflavones. Although 5,7,4′-trihydroxy-3′-methoxyisoflavone and 5,7,3′-trihydroxy-4′-methoxyisoflavone have been isolated from several plant parts, such as the stems of *Erycibe expansa* [14] and the branch wood of *Andira surinamensis* [10], they are rare in Nature. Development of an easy method for producing the two methoxyisoflavones would open the research field to exploring the bioactivity of these two compounds in the future. In addition, we found that 5,7,4′-trihydroxy-3′-methoxyisoflavone exhibited potent anti-proliferative activity on mouse B16 melanoma cells, with an IC_50_ value of 68.8 μM, but showed no significant growth inhibition on mouse normal fibroblast cells, even at 350 μM concentration (Table 1). The preliminary findings highlight the potential use of 5,7,4′-trihydroxy-3′-methoxyisoflavone for its anti-melanoma activity.

In addition to 5,7,4′-trihydroxy-3′-methoxyisoflavone, in the present study the biotransformation precursor genistein was also found to display potent cytotoxicity against B16 melanoma cells (Table 1). The result was consistent with that reported by Record et al. [15]. In fact, genistein also shows potent cytotoxicity to other melanoma cells [16]. Although genistein possesses potent anti-melanoma activities, however, it suffers from the apparent drawback of low bioavailability [17]. It has been reported that methylation of free hydroxyl groups in flavonoids dramatically increases their metabolic stability and enhances cell membrane transport, leading to facilitated absorption, and this greatly increases oral bioavailability [18,19]. Therefore, the methoxygenistein derivative 5,7,4′-trihydroxy-3′-methoxyisoflavone produced in the present study may possess more potential in cancer therapy than its precursor genistein. However, more detailed studies need to be done in the future to confirm this.

Regarding substrate specificity, both enzymes used in the present study possess a broad substrate spectrum. In addition to the natural substrates tyrosine and l-3,4-dihydroxyphenylalanine (l-DOPA), *B. megaterium* tyrosinase has been proven to transform the isoflavone aglycones daidzein and genistein, the isoflavone glycosides daidzin and genistin, and the stilbene resveratrol [6,7]. The range of the chemical structures of the catalyzed substrates is quite large and includes a single benzene ring (tyrosine and l-DOPA), two benzene rings (resveratrol), or three rings (flavonoids). *S. peucetius O*-methyltransferase belongs to the class I *O*-methyltransferases (also known as caffeoyl coenzyme A OMTs or CCoAOMTs), which are involved in methylation of phenolic hydroxyl residues [20]. The enzyme has been shown to catalyze many flavonoids, including flavonols (quercetin, rutin), flavones (7,8-dihydroxyflavone, luteolin), a flavanone (naringenin), and isoflavonoids (daidzein, 8-hydroxydaidzein, and formononetin) [8,19]. Based on the results of the previous studies, it is highly possible that the two-step biotransformation process developed in the present study could also be applied to other flavonoids or even simple phenolic compounds. Currently several simple phenolic or flavonoid precursors, including daidzin, genistin, daidzein, resveratrol, apigenin, naringin, naringenin, morin, arbutin, hordenine, and synephrine, are being tested by the two-step biotransformation system in our laboratory. Through the two step biotransformation process developed in the present study, many methoxyflavonoids derivatives could be produced in the future. In addition to the actual production of novel methoxyflavonoids, research on the multiple bioactivities of the produced methoxyflavonoids would also be an interesting future area of study.

## 4. Materials and Methods

### 4.1. Microorganisms, Animal Cells, and Chemicals

Both recombinant *E. coli* expressing *B. megaterium* tyrosinase and recombinant *E. coli* expressing *S. peucetius O*-methyltransferase were constructed in our laboratory per our previous work [7,8]. We purchased both mouse B16-F10 melanoma cells BCRC 60031 and mouse normal fibroblast cells BCRC 60203 from the Bioresources Collection and Research Center (BCRC, Food Industry Research and Development Institute, Hsinchu, Taiwan). Genistein, isopropyl-β-d-thiogalactopyranoside (IPTG), DMSO, SAM, and 3-(4,5-dimethylthiazol-2-yl)-2,5-diphenyltetrazolium bromide (MTT) were purchased from Sigma (St. Louis, MO, USA). The other reagents and solvents used were of high quality and were purchased from commercially available sources.

### 4.2. Preparation of Biocatalyst

The lyophilized recombinant *E. coli* cells were used as the biocatalyst in this study, and we prepared these cells based on our previous work [7], as described briefly below. The recombinant cells were cultured in 100 mL of Luria-Bertani (LB) medium containing 100 μg/mL of ampicillin with 180 rpm shaking at 37 °C. As the optical density at 600 nm reached 0.6, 0.1 mM of IPTG was added to induce gene expression. The cells were continuously cultivated at 18 °C for another 24 h. At the end of the cultivation, the cells were harvested and washed once with phosphate-buffered saline (PBS, pH 6.8). Cell pellets from the washed cells were lyophilized by a freeze dryer.

### 4.3. Biotransformation of Both Hydroxylation and Methylation

Genistein (200 mg, 100 mg/mL in DMSO) was added to reaction mixture (100 mL) containing 500 mM of borate pH 9.0, 10 mM of ascorbic acid, and 20 mg of the lyophilized recombinant *E. coli* expressing *B. megaterium* tyrosinase to start the hydroxylation reaction. The reaction was run at 50 °C and 200 rpm for 1.5 h in an incubator. At the end of the reaction, 1 M HCl (20 mL) was added to stop the reaction. Then, ethyl acetate (120 mL) was added to extract the isoflavones from the reaction mixture. The ethyl acetate fraction was dried under vacuum. The dried mass (373 mg) was dissolved in DMSO (100 mg/mL), analyzed by UPLC, and added into another 100 mL of reaction mixture containing 1 mM of SAM and 20 mg of the lyophilized recombinant *E. coli* expressing *S. peucetius O*-methyltransferase to start the methylation reaction. The reaction was run at 40 °C and 200 rpm for 24 h in an incubator. At the end of the reaction, ethyl acetate (100 mL) was added to extract the isoflavones from the reaction mixture. The ethyl acetate fraction was dried under vacuum. The dried mass was dissolved in 100 mL of 50% (*v*/*v*) methanol, analyzed by UPLC, and used for purification of metabolites.

### 4.4. UPLC Analysis

Biotransformation mixtures were analyzed with a UPLC system (Acquity UPLC H-Class, Waters, Milford, MA, USA) equipped with an analytic C18 reversed-phase column (Acquity UPLC BEH C18, 1.7 μm, 2.1 mm i.d. × 100 mm, Waters). The operation conditions included a gradient elution using water (A) containing 1% (*v*/*v*) acetic acid and methanol (B) with a linear gradient for nine min with 35% to 80% B and for another one minute with 80% to 85% B at a flow rate of 0.3 mL/min, injection volume of 0.2 μL, and detection of the absorbance at 260 nm.

### 4.5. Purification and Identification of Biotransformation Products

The final reaction mixture obtained from the above biotransformation (in 100 mL of 50% methanol) was centrifuged at 10,000 rpm and filtered with a 0.22 μm nylon membrane under vacuum. Then, the filtrate was injected into a preparative YoungLin HPLC system (YL9100, YL Instrument, Gyeonggi-do, Korea) equipped with a preparative C18 reversed-phase column (Inertsil, 10 μm, 20.0 mm i.d. × 250 mm, ODS 3, GL Sciences, Eindhoven, The Netherlands) for purification of the biotransformation products. The operational conditions for the preparative HPLC analysis were the same as those in the UPLC analysis. The elution corresponding to the peak of the metabolite in the UPLC analysis was collected, condensed under a vacuum, and then crystallized by freeze drying. Finally, 27.9 mg of compound (**1**) and 33.3 mg of compound (**2**) were obtained. The structures of the compounds were confirmed with NMR and mass spectral analysis. The mass spectral analysis was performed on a Finnigan LCQ Duo mass spectrometer (ThermoQuest Corp., San Jose, CA, USA) equipped with electrospray ionization (ESI). ^1^H- and ^13^C-NMR, HMQC, HMBC, COSY, and NOESY spectra were recorded on an AV-700 NMR spectrometer (Bruker Corp., Billerica, MA, USA) at ambient temperature. Standard pulse sequences and parameters were used for the NMR experiments, and all chemical shifts were reported in parts per million (ppm, δ).

*5,7,4′-Trihydroxy-3′-methoxyisoflavone* (**1**). ^1^H-NMR (DMSO-*d*_6_) δ: 8.34 (1H, s, H-2), 7.13 (1H, d, *J* = 2.1 Hz, H-2′), 6.98 (1H, d, *J* = 8.1, 2.1 Hz, H-6′), 6.82 (1H, d, *J* = 8.1, H-5′), 6.37 (1H, d, *J* = 2.2 Hz, H-8), 6.21 (1H, *J* = 2.2 Hz, H-6), 3.79 (3H, s, MeO-3′); ^13^C-NMR (DMSO-*d*_6_): 180.1 (C-4), 164.8 (C-7), 162.0 (C-5), 157.6 (C-9), 154.1 (C-2), 147.3 (C-3′), 146.7 (C-4′), 122.3 (C-3), 121.7 (overlap, C-1′, C-6′), 115.3 (C-5′), 113.3 (C-2′), 104.3 (C-10), 99.1 (C-6), 93.8 (C-8), 55.7 (OCH_3_). 

*5,7,3′-Trihydroxy-4′-methoxyisoflavone* (**2**). ^1^H-NMR (DMSO-*d*_6_) δ: 8.28 (1H, s, H-2), 7.02 (1H, d, *J* = 2.1 Hz, H-2′), 6.96 (1H, d, *J* = 8.3, H-5′), 6.93 (1H, dd, *J* = 8.3, 2.1 Hz, H-6′), 6.32 (1H, d, *J* = 2.1 Hz, H-8), 6.17 (1H, d, *J* = 2.1 Hz, H-6), 3.79 (3H, s, MeO-4′); ^13^C-NMR (DMSO-*d*_6_) δ: 179.9 (C-4), 165.8 (C-7), 162.0 (C-9), 157.7 (C-2), 154.0 (C-4′), 146.1 (C-3′), 123.5 (C-1′), 122.0 (C-3), 119.8 (C-6′), 116.4 (C-2′), 112.0 (C-5′), 103.9 (C-10), 99.4 (C-6), 93.9 (C-8), 55.7 (OCH_3_).

### 4.6. Determination of Cell Viability

Cell viability was determined as previously reported [8] and described briefly below. Dulbecco’s modified Eagle’s medium (DMEM) containing 10% (*v*/*v*) fetal bovine serum was used to cultivate the tested cells, which were incubated at 37 °C in a humidified, CO_2_-controlled (5%) incubator. After one day of incubation, the cells were treated with tested drugs for another two days. The drug-treated cells were then harvested and the cell viability was measured according to our previous study [8].

## 5. Conclusions

The present study first developed a bio-production process for 5,7,4′-trihydroxy-3′-methoxyisoflavone (**1**) and 5,7,3′-trihydroxy-4′-methoxyisoflavone (**2**), where genistein was firstly biotransformed to 3′-hydroxygenistein by recombinant *E. coli* that expressed *B. megaterium* tyrosinase and the produced 3′-hydroxygenistein was then biotransformed to 5,7,4′-trihydroxy-3′-methoxyisoflavone and 5,7,3′-trihydroxy-4′-methoxyisoflavone by recombinant *E. coli* that expressed *S. peucetius O*-methyltransferase. Moreover, the results of the present study also showed the specific potent anti-melanoma activity of 5,7,4′-trihydroxy-3′-methoxyisoflavone in cultured mouse cells.

## Figures and Tables

**Table 1:** Cell survival ^1^ of the studied isoflavones toward mouse B16 melanoma cells and mouse normal fibroblast cells.

Cells	Melanoma			Fibroblast		
Isoflavone	Genistein	1	2	Genistein	1	2
**Control**	100 ± 3.21	100 ± 4.48	100 ± 6.64	100 ± 4.12	100 ± 3.41	100 ± 0.22
**11 μM**	102.41 ± 4.52	99.15 ± 7.42	92.67 ± 2.27	97.81 ± 4.67	103.68 ± 3.57	102.42 ± 4.97
**22 μM**	77.55 ± 3.55 *	80.77 ± 4.53 *	83.57 ± 4.43 *	102.33 ± 3.76	102.09 ± 3.86	99.28 ± 0.42
**44 μM**	55.28 ± 2.99 *	57.9 ± 5.18 *	82.79 ± 3.39 *	98.91 ± 3.41	94.69 ± 3.71	93.58 ± 2.43 *
**88 μM**	44.41 ± 1.92 *	40.38 ± 1.69 *	81.72 ± 2.78 *	96.7 ± 2.89	97.69 ± 2.28	89.77 ± 1.18 *
**175 μM**	41.72 ± 3.11 *	32.05 ± 0.9 *	74.19 ± 0.29 *	92.11 ± 3.12	102.09 ± 3.86	84.26 ± 1.16 *
**350 μM**	39.81 ± 1.91 *	33.54 ± 2.89 *	53.86 ± 3.00 *	85.77 ± 1.98 *	89.21 ± 4.99	71.70 ± 1.00 *
**IC_50_^2^ (μM)**	70.3	68.1	420.3	N.D. ^3^	N.D.	N.D.

^1^ The mean (*n* = 3) is shown, and the S.D. is represented by error. A value of *p* < 0.05 (*) from a student’s *t*-test analysis by comparing the data with that of the control was considered to be statistically significant. ^2^ The IC_50_ values represent the concentrations required for 50% inhibition on the cell growth. ^3^ N.D. represented not determined.
